# Perspectives on vulnerability from the narratives of older migrants

**DOI:** 10.1007/s00391-024-02328-x

**Published:** 2024-06-21

**Authors:** Anna-Christina Kainradl

**Affiliations:** Center for Interdisciplinary Research on Aging and Care (CIRAC), University of Graz, Schubertstraße 23/1, 8010 Graz, Austria

**Keywords:** Aging, Migration, Intersectionality, Ethics, Autonomy, Altern, Migration, Intersektionalität, Ethik, Autonomie

## Abstract

**Background:**

Older migrants are considered a vulnerable population group in many ways. Marginalization and social exclusion lead to unequal opportunities for social participation.

**Aim:**

In order to break down barriers for older migrants, the perspectives of people with migration biographies should be given greater consideration.

**Material and methods:**

To this end, the results of an explorative intersectional ethical analysis of care narratives of older migrants are discussed in the light of aging studies research. The focus is on the ethical analysis of five guided interviews with older migrants between 65 and 80 years old, who have migrated from different countries in southeastern Europe.

**Results and discussion:**

In contrast to the prevalent expert perspective, the narratives of the older migrants interviewed revealed not only resistance to vulnerabilization but also multiple negotiations of autonomy and dependency. By making ambivalent narrative and action strategies visible and linking them to narratives of intergenerational care relationships, the significance of care-ethical interpretations of vulnerability and characterization of vulnerability as “a universal, inevitable, and anthropological feature of humanity resulting from the embodied, finite, and socially contingent structure of human existence” [4] can be demonstrated.

## Abstract (de)

**Hintergrund:**

Ältere Migrant:innen werden in vielfacher Weise als vulnerable Bevölkerungsgruppe betrachtet. Marginalisierung und soziale Exklusion führen zu ungleichen Chancen sozialer Teilhabe.

**Ziel der Arbeit:**

Um Barrieren für ältere Migrant:innen abzubauen, soll der Perspektive von Menschen mit Migrationsbiografien stärkere Berücksichtigung zuteilwerden.

**Material und Methoden:**

Dazu werden Ergebnisse einer explorativen intersektionalen Ethikanalyse von Care-Narrativen älterer Migrant:innen im Lichte alternswissenschaftlicher Forschung diskutiert. Im Zentrum steht die ethische Analyse fünf leitfadengestützter Interviews mit älteren Menschen mit Migrationsgeschichte im Alter zwischen 65 und 80 Jahren, die aus verschiedenen Ländern Südosteuropas migriert sind.

**Ergebnisse und Diskussion:**

Im Gegensatz zur vorherrschenden Expert:innenperspektive lassen sich in den Narrativen der interviewten älteren Migrant:innen nicht nur Widerständigkeit gegenüber Vulnerabilisierung, sondern auch vielfältige Aushandlungen von Autonomie und Abhängigkeit erkennen. Indem ambivalente Erzähl- und Handlungsstrategien sichtbar gemacht und mit Erzählungen intergenerationeller Care-Beziehungen verbunden werden, können care-ethische Interpretationen von Vulnerabilität sowie Charakterisierungen von Vulnerabilität als „a universal, inevitable, and anthropological feature of humanity resulting from the embodied, finite, and socially contingent structure of human existence“ [4] in ihrer Bedeutung aufgezeigt werden.

## Introduction

In many respects, older people who migrated to another country are considered a vulnerable population group [7]. In contrast to the prevalent expert perspectives [25], this article draws attention to how older migrants themselves deal with topics related to vulnerability. In dealing with autonomy and dependency as well as in their narratives of intergenerational relations the interviewed older migrants show multiple and ambivalent understandings of vulnerability. This article therefore discusses these understandings as well as the potential that an intersectional ethics analysis has for care narratives of older migrants to shed light on their negotiations of vulnerability. In this context five qualitative interviews with older migrants provide insights into their perspectives on vulnerability in health issues. The meaning and coping strategies of older migrants and their perceptions and assessments of vulnerability can provide further insights for the theoretical discussion of vulnerability.

## Background

Older migrants represent a rapidly growing population group, both in the overall population [29] and in care contexts and care practice in Austria [21]. This population group differs not only in how they migrated and how they refer to their migration experience. They are heterogeneous in many respects, such as biographically, socioeconomically, educationally and ethnically [21] Labor migrants who came to Austria in the 1970s, make up a large part of this group. The growth can be seen in the groups of people with non-Austrian citizenship and those born in countries other than Austria, both in age groups of 45–54 years and 65+ years [29].

The very choice of the term for these people, reflects the great heterogeneity of this group with respect to their biographies, origins, their subjective approaches to their own migration history and their life course [5, 8]. Given the significance and ambivalence of characterization of these people, the group of people with migration history selected for this article are referred to as older migrants. The term chosen is extremely common in the field of ethics and health research and has the advantage of implicitly recognizing the heterogeneity of this group, also in its subjective references, very well.

Both research in aging and in migration studies face the challenge of adequately analyzing the interplay between the inequality factors of migration and age on an individual and structural level as well as the interactions between individuals and social structures. Statistically, these people are categorized differently, according to the countries in which they were born, their citizenship, or their language. Even though there are generally still large gaps in the research on older people with a history of migration [2], certain groups of older migrants can be considered comparatively well-researched in terms of their vulnerability to health issues.

In both health science and ethical research, this population group is recognized as being vulnerable in many respects [23]. For example, Reinprecht et al. [21] emphasized in their research on the care perceptions of older migrants: “The intertwining of variables such as age, gender, social status, and foreign origin increases the potential risk of social vulnerability. The experiences of social marginalization (low income, inadequate housing, scarce educational resources (language skills), working conditions that are detrimental to health, lack of social recognition, and discrimination) intensify in the post-professional phase” [21].

What is more, influential ethical theories, such as Beauchamp and Childress’ principlism, attribute in their reflections on vulnerable populations and individuals, the vulnerability of older migrants due to their greater exposure to exploitation and challenges in consenting to medical interventions [3] and focus as a consequence on the protective measures. It becomes clear that it is not only the research on older migrants itself that is very inadequate and that the potential that would be necessary for an in-depth analysis is not being utilized. Also, the studies on older migrants are dominated by the perspective of experts and relatives [25], which is often strongly deficit-orientated [2].

When older people with a migration background are asked about their experiences in the Austrian healthcare system, topics that are discussed in scientific research in the field of vulnerability appear in their narratives. They deal with questions of autonomy and dependence, the search for and understanding of information, trust, and organization of relationships. Therefore, they cover aspects which Schweda categorizes as damage-related and interest-related vulnerability [24]; however, how these topics are narrated broadens the view from the particular endangerment of this population group to an understanding of vulnerability as an ambivalent interpreted basis for care relationships.

## Method

The population group chosen for the explorative intersectional ethics analysis was the group of older people who come from labor migration countries, i.e., from countries in southeastern Europe, as this is the largest group of older people with a history of migration in Austria [20]. Most members of this group have guaranteed access to the healthcare system due to their early migration and many years of work in Austria; therefore, questions of legally secured healthcare and, from an ethical perspective, questions of the fundamental right to it, do not need to be addressed further.

The chronological minimum age of 60 years was selected, which takes into account both the World Health Organization (WHO) definitions [27] and the subjective experience of the post-professional phase of life with the associated individual and social attributions [31]. In this study 5 migrants aged between 65 and 80 years, who differ in terms of the country of origin, their language skills, but also their housing and care situation, were recruited as interview partners. They were chosen to gain insights into different experiences in the Austrian healthcare system through these differences representing central topics in the research of inequity in health issues.

The concept of intersectionality was chosen as the theoretical lens for analysis. This concept goes back to the lawyer Kimberlé Crenshaw and the American civil rights movement. It is rooted in the refusal of two court rulings to legally recognize the interaction of two factors of inequality (gender and race), as well as in Crenshaw’s analysis of US antidiscrimination legislation and was developed in the 1980s. For the analysis of the narratives of older migrants, its potential proves to reflect on individual differentiations, their representations as well as their structural conditions of origin and to focus on their dynamic interactions [28].

The perspectives of older migrants, their perceptions of health and illness, in addition to their experiences in the Austrian healthcare system, were collected using qualitative individual interviews. The thematically semi-structured guideline-based individual interviews [13] were analyzed using the qualitative content analysis method according to Kuckartz [16]. The central themes of the interviews were first thematically analyzed and compared. As part of the further analysis, the deductive categories of age, migration and health derived from the thematic analysis and the intersectionality discourse were complemented by inductively developed subcategories. The interactions between age and other categories of difference were then analyzed and related to each other from an intersectional perspective. By analyzing the multiple entanglements of age, it was possible to examine the negotiations of autonomy and justice that took place in a more nuanced way and to look for possible indications of their representations, as well as their structural conditions [28]. All interview quotes were anonymized for the purposes of this article and translated into English by the author.

## Findings

### Negotiating vulnerability in intergenerational relationships

The narratives of older migrants often address issues that are associated with vulnerability in research. In the narratives on age and migration, questions of independence and dependence, the search for and understanding of information, as well as questions of trust and the organization of relationships are central themes. Concerning the underlying inequality factor of age, which is often regarded in research as a characteristic of particular vulnerability [17], older migrants position themselves differently.

In the narratives of the older migrants interviewed, their own age is not described as a chronological age. The self-portrayals and age attributions tie in with findings that have been uncovered in aging studies on the “ageless self” [15, 26] and on the “transition to dependent old age anticipated as a break” [12, which is only attributed to others who already entered the group of the undesirable fourth age [11]. The “ageless self” is utilized for one’s own self-presentation and life, while the “transition to dependent old age anticipated as a break” is used to construct the group of older people in othering processes. It is only with “illness, the need for care, and the loss of a self-determined lifestyle” [1] that the transition to “real old age” occurs, which is true even for a group of people to whom, however, according to Graefe et al. even very old people rarely feel they belong “as long as the ability to shape their lives—within whatever limited framework—is still given” [12].

In the context of intergenerational relationships, however, there is an additional perspective, that is mentioned: “My mum, my mum, she was a teacher and always she was unhappy. […] and it was really exhausting” (Interview 5, position 57; own translation). This led to a critical assessment and the taking up of social images of old age with the implied accusations of burdening younger generations [14]. The “burden narrative” [6] in particular addresses the desire not to be a burden on one’s own children and to work on one’s own independence. This interpretation is also supported by the fact that several interviewees proudly emphasized not being a burden on the children and striving for independence. They expressed that they do consider others being part of the group of older people who need care and support: “Many people need help for many things […] People need contact and support” [Interview 5, position 8; own translation]. The implied burden that the group of older people could potentially represent, results from their need of help and influences the relations to other generational groups. It reflects the findings of aging studies that address both the binary construction of population groups and the conflicts that arise from the characterization of “a rapidly growing population of needy, relatively affluent individuals whose collective dependence is straining the economies of Western welfare states and creating excessive tax burdens for younger generations” [14].

This is explicitly emphasized by an older migrant who described a conversation with her own daughter during a hospital stay: “And I don’t want to burden her.” (Interview 4, position 56; own translation). Her desire not to be a burden on her daughter is mentioned several times during the interview. Particularly when she talks about a visit in a hospital, and therefore being part of a group that requires care, this wish is also upheld in response to the daughter’s requests and independence is asserted.

In addition to these references to the support provided by the children, their own care activities are described in great detail, underpinned by positive emotionality and an emphasis on the own effort in bringing up the children: “My children are my biggest success.” (Interview 5, position 59; own translation). These caring activities are interpreted as a source of satisfaction and quality of life in intergenerational care relationships; however, the importance of intergenerational relationships is also described as positive and central to one’s own good life in cases where there is no active care and the children and grandchildren endeavor to maintain a relationship with the older migrants, for example, by coming to visit to celebrate a birthday. According to Paal and Bükki it can be assumed that intergenerational relations are “perceived as a very potent resource of comfort and support” [19]. This means that the focus of these care narratives is not so much on the reciprocity of the caring relationship between generations and on the proof of one’s own usefulness vis à vis agist accusations of being a burden. Rather they show an ambivalence between the taken for granted intergenerational “fields of solidarity” [9] and the fear of being a burden.

### Negotiating vulnerability and autonomy

The category of age, which is often associated with vulnerability in health science research, can be linked to other categories thanks to the intersectional analysis. Age can also be found in the interviews in a life course perspective: age is referred to insofar as one looks back on the life already lived, their experiences, conclusions, and consequences. In contrast to the description of other older people and the juxtaposition of the younger and older generations, age is interpreted more ambivalently, because “[t]his is all age. Every wrinkle has something. Every wrinkle has something to tell” (Interview 4, position 144; own translation). Health problems that are attributed to age are described as minor: “I have some really small problems with my knees” (Interview 1, position 192; own translation) or mentioned as the normal results of a strenuous working life. From an intersectional perspective this reveals a strong link and changing references to other categories of inequality, such as socioeconomic factors in the self-portrayals of the interviewed older migrants.

In the narratives of older migrants interviewed, their own vulnerability was addressed by referring to questions of independence and dependence, the search for and understanding of information, and questions of trust and the organization of relationships. Older migrants deal with their own vulnerability in close coordination with their ability to plan and shape their own lives in an informed manner. Their own knowledge is also confidently anchored in relation to medical advice. The interviewed older migrants reported on their own divergent decisions that led them not to follow medical advice and reflect on the reasons behind them: “Now, for example, he told me, my orthopedic surgeon, I should have surgery on my left hip. Okay. But to be honest, I think because it’s still possible. I wouldn’t like that. He said: ‘But what about when you get older? And then it comes and maybe it won’t be too late?’ I don’t care. One day I have to die, I don’t know what I have to die from, but I hope it won’t be so bad that I have to die from it” (Interview 4, position 68; own translation).

The trade-offs between the consequences and current quality of life are repeated and it is emphasized that the decision in favor of medical advice can also be made later. Their own expertise in this ethical trade-off process is not called into question and highlighted against medical advice. Older migrants are obviously also orientated towards an ideal of autonomy that has long been criticized by feminist theorists as “one-sidedly rationalistic, individualistic (egoistic), unrelated and oriented towards the male model of life” [22]. This orientation also clearly emphasizes the ideas of the good life as being associated with “quality of life […] closely linked to the claim to autonomy of (European) modernity” [20]; however, autonomy is lived and interpreted by the older migrants interviewed differently than autonomy in the societies of the global north. It is understood more relationally and reflects the critique of feminist ethicists [22]. This becomes clear, for example, in a comment by K., who emphasized her mental illness and stressed in her stories: “I can fix myself on my own” (Interview 5, position 4; own translation), only to then naturally report on her colleagues and their support and conclude the story with the sentence “[…] I am always in contact” (Interview 5, position 4; own translation). This highlights that agency, expertise, autonomy and dependency as central topics in the field of vulnerability are negotiated in the care narratives of the interviewed migrants in multiple and ambivalent ways.

## Discussion

The older migrants interviewed tell their own stories of vulnerability in various and ambivalent ways; between their own independence and expertise along with their organization of intergenerational relationships and adherence to the ideal of autonomy. Even if vulnerability is ascribed to them in many respects, the way in which values, such as responsibility and autonomy are negotiated initially, reveals resistance to vulnerabilization [10]. It is seen as a threat to the societal ideal of autonomy and consequently the rejection of social images of old age as a dependency and burden for younger generations. An intersectional analysis that focuses on the dynamic interpretations and entanglements of age as well as on dynamics between levels of structure, representation, and identity [27] enables further narrative and action strategies to be made visible. It links narratives of intergenerational relations, of self-confident expertise, and of relational autonomy. These findings can be taken up with care-ethical interpretations of vulnerability and characterization of vulnerability as “a universal, inevitable, and anthropological feature of humanity resulting from the embodied, finite, and socially contingent structure of human existence” [4]. In this way, the concept of vulnerability can be used to recognize both potentials and challenges without “transforming challenging life events into opportunities for coping and bounding back” [30]. In the narratives of older migrants interviewed, their own vulnerability and strength, their own integration into relationships, and their own autonomy do not contradict each other. Sometimes they are in a tense relationship, but often they are also uncommented on as if their co-existence and entanglement are obvious.

## Conclusion

Against the background of an intersectional ethics analysis, first insights into the multiple interpretations and entanglements of age can be clearly shown. Structural factors and societal images of age have a dynamic effect on the narratives of older migrants and on their negotiations of questions of autonomy and dependence, the search for and understanding of information, and questions of trust and the shaping of relationships. From an intersectional perspective, it can therefore be concluded that vulnerability is not only addressed as a negative threat to one’s own independence and informed consent. Rather, these are naturally supplemented in the interviews by more relational conceptualizations of autonomy and intergenerational inclusion. From a care-ethics perspective, these narratives therefore show that “vulnerability and autonomy are not to be theorized in opposite terms” [18] but refer to each other and are connected.

## Practical conclusion

- Both a deficit-oriented perspective and the clear categorization of older migrants as a vulnerable group, should be questioned from the perspective of those affected and should be further developed with the help of the narratives of older migrants themselves.
- It is proposed that the diverse, intersectional entanglements between various categories of difference and inequality should be taken into greater consideration at different levels of the healthcare system.
- The article poses the question of which interpretations and extensions enable an intersectional ethics analysis of the narratives of older migrants. Specific places and opportunities to better recognize the narratives of older migrants and incorporate them into the design of the Austrian healthcare system should be developed.

## Declarations

All procedures performed in studies involving human participants or on human tissue were in accordance with the ethical standards of the institutional and/or national research committee and with the 1975 Helsinki declaration and its later amendments or comparable ethical standards. Informed consent was obtained from all individual participants included in the study.
